# High-performance parallel tandem MoTe_2_/perovskite solar cell based on reduced graphene oxide as hole transport layer

**DOI:** 10.1038/s41598-022-25015-6

**Published:** 2022-11-28

**Authors:** Mohammad Gholipoor, Nasrin Solhtalab, Mohammad Hosein Mohammadi

**Affiliations:** 1 Department of Energy Science, Sungkyunkwan University (SKKU), Suwon, Republic of Korea; 2 Department of Electrical and Computer Engineering, Tarbiat Modares University (TMU), Tehran, Iran

**Keywords:** Nanoscience and technology, Optics and photonics

## Abstract

Recently, the impressive achievements accomplished in multijunction (tandem) perovskite solar cells have triggered a huge research effort to boost their performance. Here, using a three-dimensional (3D) finite element method (FEM) technique, we propose and investigate a parallel tandem PSCs consisting of two absorbing layers of MoTe_2_ and CH_3_NH_3_PbI_3_ with cascaded bandgaps to more efficiently use the near-infrared (NIR) solar spectrum. Endowed with a bandgap of about 1 eV, the MoTe_2_ layer in conjunction with a CH_3_NH_3_PbI_3_ layer is able to broaden the light absorption range of structure beyond the wavelength of 800 nm, up to 1200 nm. In addition to this, the MoTe_2_ material can not only appreciably harvest light even with a thickness as low as 20 nm due to their high absorption coefficient, but also make a perfect band alignment with the CH_3_NH_3_PbI_3_ layer. As a result, the proposed multijunction PCS yields a high power conversion efficiency (PCE) of 18.52% with a V_OC_ of 0.83 V, J_sc_ of 26.25 mA/cm^2^, and FF of 0.84, which is considerably greater than its corresponding single-junction PSCs with PCE, V_OC_, J_sc_, and FF of, 14.01%, 1.14 V, 15.20 mA/cm^2^, and 0.81, respectively. Furthermore, to mitigate the V_OC_ loss caused by the low bandgap of MoTe_2_, we demonstrate an increase in V_OC_ from 0.84 to 0.928 V and in PCE from 18.52% to 20.32%, when we replace a reduced graphene oxide (rGO) layer with Spiro-OMeTAD layer as a hole transport layer (HTL).

## Introduction

Organic–inorganic metal hybrid perovskites have been consistently arousing extraordinary research interest in the photovoltaic community owing to their exceptional semiconductor properties such as facile fabrication process, long diffusion length^1^, long carrier lifetime^2^, panchromatic absorption of light^3^, etc. To date, the maximum power conversion efficiency (PCE) achieved in single-junction perovskite solar cells (PSCs) has been as high as 25.5%^1^. So as to further enhance the PCE constrained by the Shockley–Queisser (SQ) limit, some different strategies were pursued, namely, the carrier multiplication effect to harvest the additional energy (hυ-E_g_) of photons with energy larger than bandgap (E_g_)^4^ and multijunction absorbers to harvest photons with energy smaller than E_g_^5^. Whereas it still is impractical and elusive to gain the PCE via carrier multiplication phenomena, multijunction (tandem) PSCs have successfully achieved the PCE as large as 29.15%^6^. However, inspired by the achievements of counterparts of tandem PSCs, GaAs and GaInP-based multijunction solar cells which have reached a maximum PCE of 38.8%^7^, there is still a burgeoning interest in the further improvement of the multijunction PSC performance. This has spurred the search for new materials and architectures for multijunction PSCs.

Semiconducting transition metal dichalcogenides (TMDs), including MoS_2_, MoSe_2_, MoTe_2_, WS_2_, and WSe_2_, are emerging as highly impressive absorbers for solar cells owing to their ultrahigh absorption coefficients^8^, mechanical flexibility^9^, high carrier mobility^10^, together with an ideal bandgap for photovoltaic applications^8^. Notably, a TMD layer thinner than 20 nm is able to absorb light even ten times larger than well-known direct bandgap semiconductors^8^. While the TMDs, especially MoS_2_, have been widely employed as carrier transport layers (HTLs) in the PSCs^11,12^, there is no report of deriving a benefit from the TMDs absorption capacity in order to improve the light absorption efficiency in PSCs. Although most TMDs have almost the same bandgap magnitude as perovskites, bulk MoTe_2_ with a bandgap of around 1 eV would be a complementary absorbing material for perovskite to harvest the near-infrared (NIR) range of sunlight. The strong NIR absorption capability of MoTe_2_, along with the absence of dangling bonds at its surface, a property of TMDs which originate from their weak van der Waals (vdW) interlayer interaction, underpin MoTe_2_ aa a suitable candidate to be heterostructured with perovskite materials for tandem solar cells^13,14^. Experimentally, the cost-effective chemical and mechanical exfoliation methods available allow for uniform and homogeneous thin MoTe_2_ film preparation^15,16^. Thus, it would be more valuable to explore the exploitation of MoTe_2_ materials as a supportive absorbing layer, to benefit from the MoTe_2_ absorption.

Herein, we numerically present and propose a Planar type of parallel multijunction PSCs with an absorbing region made of a thin MoTe_2_ and CH_3_NH_3_PbI_3_. The main device is composed of ITO/TiO_2_/CH_3_NH_3_PbI_3_/MoTe_2_/Spiro-OMeTAD/Ag layers, a configuration that was likewise fabricated with MoS_2_^16^. The excellently desirable band alignment of MoTe_2_ with other layers, along with its high NIR absorption capacity, remarkably paves the way for achieving higher photovoltaic efficiency. By comparing to single-junction PSCs, the proposed device yields an increase in PCE from 14.01 to 18.52%. By performing an accurate numerical analysis of the MoTe_2_ thickness-dependent device performance, an optimum thickness of 25 nm was obtained, which is several orders of amplitude thinner than the previous supportive absorbing layers so far reported in multijunction PSCs^17^.

Nonetheless, it is a well-established fact that the utilization of a low band gap absorber is detrimental to the open-circuit voltage (V_OC_) of solar cells owing to the limited electron and hole quasi fermi level separation. Likewise, we have observed a reduction in V_oc_ after turning the structure into a multijunction device. In order to compensate these photovoltage losses, we replace a reduced graphene oxide (rGO) sheet with Spiro-OMeTAD as an HTL to improve the hole extraction and transportation. Outstandingly, the rGO sheet enhances the device V_OC_ and PCE up to 0.928 and 20.32%, respectively. It is noteworthy that the efficacious performance of the rGO layer as both interlayer and charge transport layer has been well proved in PSCs^18–23^.

## Basic equations and models

In this work, we employ a hybrid optical and electrical model to calculate and evaluate the presented structures. We present their traditional formulation (i.e., in the frequency domain) and then discuss the extension to the time domain. A finite element method (FEM) is used to solve the partial differential equations (PDEs).

### Optical model

**Figure 1:**
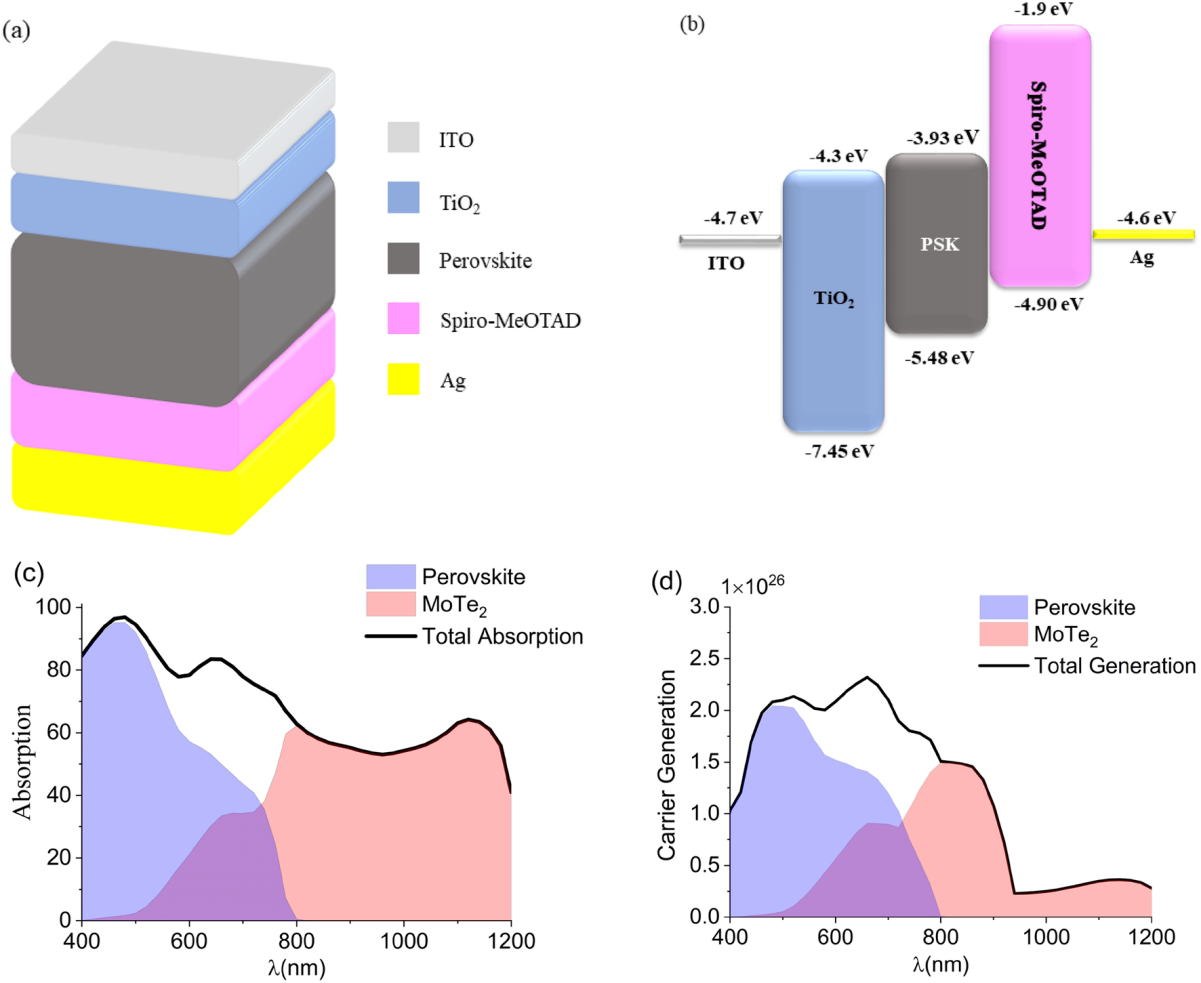
Schematic and energy diagram and device performance. (**a**) The stack structure of the basic PSC designed in this work. (**b**) the band alignment diagram and operation principle of the basic PSC. (**c**) The absorption spectra of the basic PSC, with determining the contribution of each layer. (**d**) the carrier generation rate in absorbing layers as a function of wavelength.

Figure 1A depicts the schematic diagram of our basic planar PSC scheme. From top to bottom, the structure is stacked by a transparent indium tin oxide (ITO) electrode, a compact titanium dioxide (TiO_2_) layer, a methylammonium lead iodide perovskite (CH_3_NH_3_PBI_3_) film, an N,N-di(4-methoxyphenyl)amino]-9,9′-spirobifluorene (spiro-OMeTAD) layer, and a silver (Ag) rear electrode. The incident light enters the cell from the ITO layer and is absorbed by the perovskite film to some extent. Also, the incoming light experiences a multireflection because of the rear Ag reflector which gives rise to an absorption enhancement. To quantity the interaction between electromagnetic waves and the layers, as well as the electric field (E) distribution, the Helmholtz equation (represented as follows) was solved:

$$\nabla \times \left(\nabla \times \mathrm{E}\right)-{k}_{0}^{2}{\varepsilon }_{r}E=0$$

where k_0_ is the free space wave number and ε_r_ is the dielectric constant. Clearly, to solve the above equation, one needs all the complex refractive index ($N=n=ik$) of layers as a function of wavelength. Subsequently, the E distribution obtained from solving the above Helmholtz equation enables us to compute the light absorption and carrier generation rate (G_opt_). The transfer-matrix method (TMM) is applied to estimate G_opt_ in each layer of the structure. The G_opt_ formula is as follows,

$${G}_{OPT}=\frac{{\varepsilon }^{"}{E}^{2}}{2\hslash }$$

where ℏ is the reduced Planck constant, and ε*"* is the imaginary part of the relative permittivity. As the formula obviously indicates, G_opt_ is proportional to the square of the E intensity in a certain wavelength. The total generation rate (G_tot_) can be calculated by integrating G_opt_ over an incident light wavelength bandwidth.

$${G}_{tot}={\int }_{{\lambda }_{min}}^{{\lambda }_{max}}{G}_{opt}\left(\lambda \right)d(\lambda )$$

The resulting G_tot_ is used for the input of electric model.

### Electrical model

The following well-known J–V relation is used to describe electrical characteristics of the present PSCs:

$$J\left(V\right)={J}_{dark}+{J}_{sc}={J}_{0}\left(\mathrm{exp}\left(\frac{eV}{nKT}\right)-1\right)-q{G}_{opt}({L}_{n}+{L}_{p})$$

where J_dark_ depicts the electric current of the PSCs in the absence of light illumination, J_sc_ is photocurrent, e is the electron charge, n is an ideality factor, K is the Boltzmann’s constant, and T is the temperature in kelvin. In order to calculate the currents, the following Poisson and continuity equations should be solved across the device:

$$\nabla .\left({\varepsilon }_{0}.{\varepsilon }_{r}.{\nabla }_{\varphi }\right)=-\rho$$

$$\frac{\partial n}{\partial t}=\frac{1}{q}{\nabla }_{{j}_{n}}+{G}_{n}-{U}_{N}$$

$$\frac{\partial p}{\partial t}=\frac{1}{q}{\nabla }_{{j}_{p}}+{G}_{p}-{U}_{P}$$

where ε_0_ is the permittivity of free space, *ϕ* is the electrostatic potential, *ρ* is the charge density, and q is the electron charge. Also, the J_n_ and J_p_ show the current densities arising from electrons and holes, respectively, the U_N_ and U_P_ illustrate the electron and hole recombination rates, respectively, and G_n_ and G_p_ are the electron and hole generation rates, respectively. By assuming that every absorbed photon creates one electron–hole pair, G_n_ and G_p_ are considered same to the G_tot_ obtained from the optical part.

In this study, the influence of grain boundaries and the carrier recombination at the interfaces between semiconductors are neglected. Additionally, we assume that trap-assisted recombination (SRH) inside bulk materials is the fastest and most dominant recombination mechanism in our devices.

## Results and discussion

As mentioned earlier, the reference PSC is made of ITO, TiO_2_, CH_3_NH_3_PBI_3_, spiro-OMeTAD, Ag layers, as demonstrated in Fig. 1a. The ITO, TiO_2_, CH_3_NH_3_PBI_3_, spiro-OMeTAD, and Ag layers act as the front transparent electrode, electron transport layer (ETL), absorbing layer, hole transport layer (HTL), and back electrode, respectively. Throughout this manuscript, the thickness of ITO, TiO_2_, CH_3_NH_3_PBI_3_, spiro-OMeTAD, and Ag layers are fixed at 50, 90, 200, 100, and 100 nm, respectively. Figure 1b exhibits the energy band diagram of components in the structure, approving a favorable band alignment for the electron and hole transfer across the device. Strictly speaking, the sizable valence band offset between perovskite (− 5.48 eV) and ETL (− 7.45 eV) effectively blocks the hole injection, while their conduction band is nicely aligned for collecting the excited electrons in the perovskite film. Conversely, the band alignment between CH_3_NH_3_PBI_3_ (− 3.93 eV) and Spiro-OMeTAD (− 1.95 eV) makes adequately feasible the hole transfer in the valence band, while it impedes the electron transfer in the conduction band. The input parameters, the values of energy band gap (Eg) and electron affinity (χ) of all components are chosen according to the literature^17,24,25^, and their values are indexed to the vacuum level. The black curve of Fig. 1c shows the total absorption in the reference PSC. The refractive index data of TiO_2_, CH_3_NH_3_PBI_3_, and spiro-OMeTAD are taken from the references^26–28^. The blue shaded area of Fig. 1c manifests the absorption spectra of PSK in the structure. It is clear that the perovskite layer can only absorb sunlight over 300–800 nm due to its bandgap (1.55 eV), so all NIR light is wasted. To push light absorption beyond the visible range, an ultrathin MoTe_2_ layer is placed below the perovskite film. The bulk MoTe_2_ semiconductor endowed with a small indirect bandgap of about 1.0 eV^29^ is able to extend light absorption to wavelengths up to 1200 nm, as is indicated by the pink shaded region of Fig. 1c. Besides an indirect bandgap, the bulk MoTe_2_ enjoys two dominant direct excitonic gaps, termed A and B, around 1.2 and 1.5 eV, respectively^30,31^, which specify its absorption peaks, as appeared in Fig. 1c. Subsequently, the black curve in Fig. 1d shows the total G_opt_ in the device. It confirms an efficient light absorption led to carrier generation over the NIR range. The blue and pink-shaded areas of Fig. 1d unveil the contribution of PSK and MoTe_2_ layers, respectively, to the total G_opt_. Quantitatively, the PSK and MoTe_2_ layers contribute about 61% and 39% of carrier generation, respectively. This carrier generation enhancement by the MoTe_2_ layer could be promising for cell performance improvement. Furthermore, the MoTe_2_ layer like other TMD materials can play further advantageous roles in boosting the device performance. The utility of TMDs in PSCs has been broadened to facilitate efficient carrier transport^32^, prolong the stability^16^, and so on^33^. Hence, these advantages accompanied by their low cost and easy preparation process—mechanically exfoliation and transfer into a device, affirm their effectiveness in a PSK efficiency enhancement.

In this simulation, the refractive index of bulk MoTe_2_ was obtained from the Ref^34^. Also, in all calculations, the input light source is conformed to the AM1.5G spectrum. The wavelength bandwidth is chosen from 300 to 1200 nm in a resolution of as much as 20 nm. The periodic boundary condition (PBC) is used for each side of the insulating region in the structures and the Au layer sides are set to a perfect electric conductor (PEC). The bottom and top contacts are considered ideal ohmic and Schottky with a surface recombination velocity of 107 cm/s, respectively. Furthermore, a swept mesh is applied to more precisely resolve the fields around the thin layer. Table 1 includes all optical and electrical input values used in the simulations. Herein, ε_r_ is dielectric constant, N_C_ and N_V_ are effective density of states of conduction and valence bands, μ_n_ and μ_p_ are electron and hole mobilities, χ is electron affinity, E_g_ is bandgap energy, N_A_ and N_D_ are acceptor and donor densities, and τ_n_ and τ_p_ are electron and hole lifetimes, respectively. The MoTe_2_ materials are known to be naturally P-doped^35^. In addition, in the bulk limit, the semiconducting TMDs bear photogenerated carrier lifetimes up to a few nanoseconds^36,37^.

**Table 1:** The input simulation parameters.

Parameters	TiO_2_	PSK	MoTe_2_	Spiro
ε_r_	9	6.5	13.6	10
N_c_ (cm^−3^)	1 × 10^19^	1.66 × 10^19^	1.6 × 10^19^	1.79 × 10^19^
N_v_ (cm^−3^)	1 × 10^19^	5.41 × 10^19^	2 × 10^19^	2.51 × 10^19^
µ_n_/µ_p_ (cm^2^/VS)	20/10	50/50	100/25	25/25
χ (eV)	4.1	3.93	3.9	1.9
E_g_ (eV)	3.2	1.55	1.03	3.0
NA (cm^−3^)	–	–	5 × 10^15^	5 × 10^19^
ND (cm^−3^)	5 × 10^18^	1 × 10^12^	–	–
τ_n_/τ_p_	5/2	8/8	5/5	15/15

**Figure 2:**
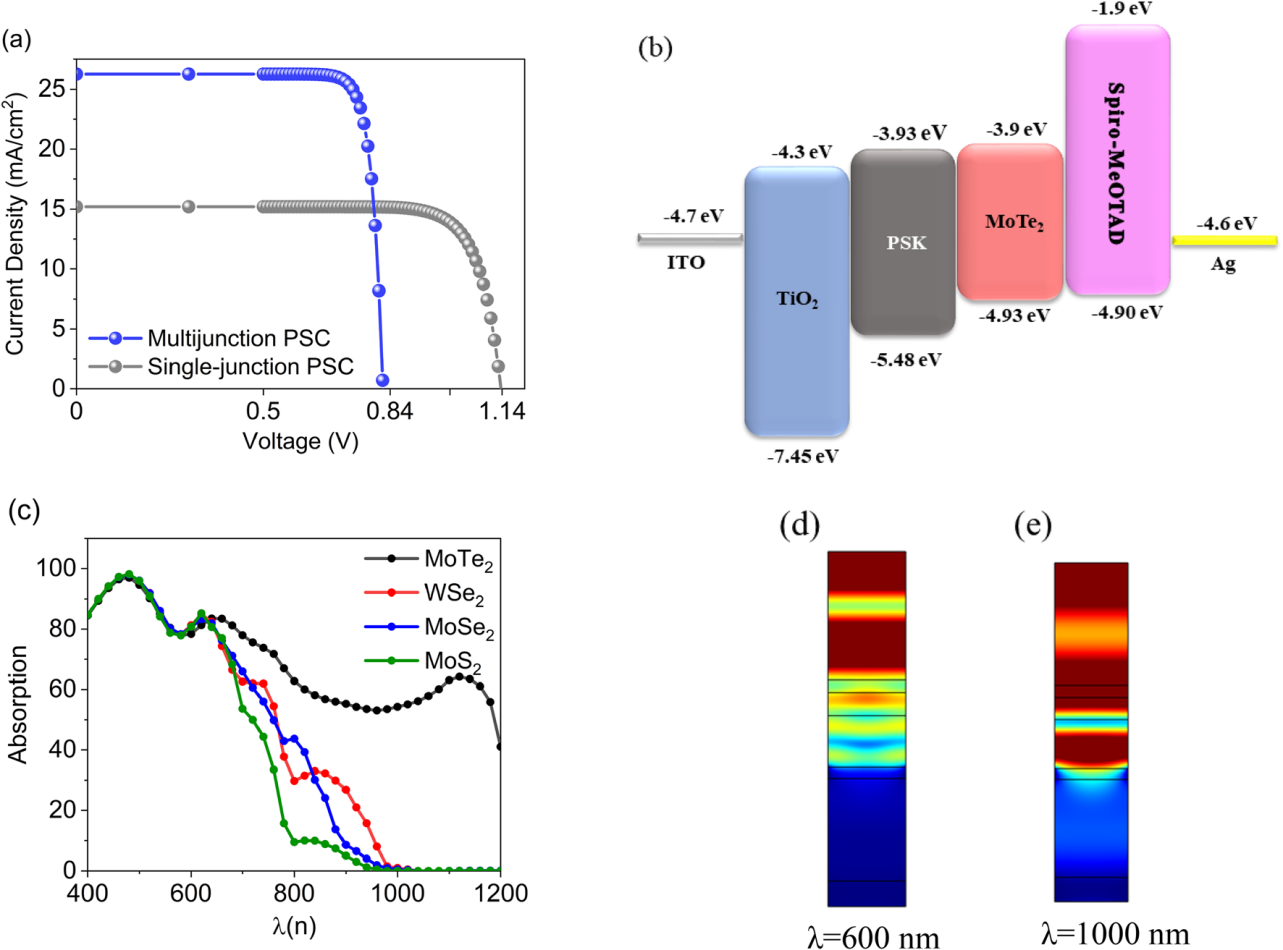
The single and multijunction PSC performance. (**a**) Current density–voltage (J–V) curve of the single PSC and multijunction PSC including MoTe_2_. (**b**) The band alignment diagram of multijunction PSC. (**c**) The absorption spectra of the structure for different TMDs including MoTe_2_, WSe_2_, MoSe_2_, and MoS_2_. (**d**) and (**e**) The normalized electric field distribution at the wavelengths of 600 and 1000 nm, respectively.

The current density–voltage (J–V) characteristics of our reference PSC under one sun condition are demonstrated in Fig. 2a. The PSC shows a PCE of 14.01%, with J_sc_ of 15.20 mA/cm^2^, V_oc_ of 1.14 V, and FF of 0.81. Benefiting the NIR light absorbed in the MoTe_2_ layer, J_sc_ considerably increases by 26.2 mA/cm^2^ in the multijunction PSC with an optimized thickness of MoTe_2_. But, the V_oc_ drops to 0.84 V due to the electron and hole quasi fermi level separation is now restricted by the MoTe_2_ bandgap. Altogether, notwithstanding the V_oc_ is destroyed after inserting the MoTe_2_ layer, the enhancement of J_sc_ is highly predominated over the V_oc_ reduction, leading to a noticeable increase in PCE from 14.01% to 18.52%. This PCE increase is also contributed by a suitable band alignment between MoTe_2_ and the perovskite layer and HTL, as indicated in Fig. 2b. Indeed, the desired band alignment between absorbing layers can effectively mitigate V_oc_ loss in multijunction PSCs as a result of charge transport improvement and charge recombination reduction^38^. In order to provide a broader perspective on the TMDs capability for light absorption, we compare the absorption spectrum of the present structure with when the MoTe_2_ layer was replaced by three other TMDs, WSe_2_, MoSe_2_, and MoS_2_, as illustrated in Fig. 2c. The refractive index and band structure parameters of WSe_2_, MoSe_2_, and MoS_2_ are obtained from the literature^34,39–41^. While all TMDs show strong light–matter interaction under light illumination, their bandgaps cover a broad range from 1–2 eV^42^. Herein, WSe_2_ and MoSe_2_ with the bandgap around 1.3 eV can absorb a wider spectrum of light compared to MoS_2_ with a bandgap of 1.45 eV. Of these, MoTe_2_ clearly is more able to absorb NIR light, making it the best choice to be cascaded with the PSK. Figure 2d,e exhibit the interaction between the light electric fields and different layers at the wavelength of 600 and 1000 nm. One can see that the MoTe_2_ layer interacts with light when the wavelength is set to 1000 nm, whereas its contribution to light absorption in the visible wavelength of 600 nm is negligible. It is also worth knowing that the utilization of TMDs in PSCs has shown successful outcomes to enhance stability^16,43^. On the other side, TMDs in each thickness can be easily prepared through environment-insensitive and non-destructive approaches such as dry or liquid-phase exfoliation^16^, then transferred by dry or wet methods. Thus, a combination of PSK materials and TMDs can potentially improve PSC performance, not only photovoltaic operation but also stability.

**Figure 3:**
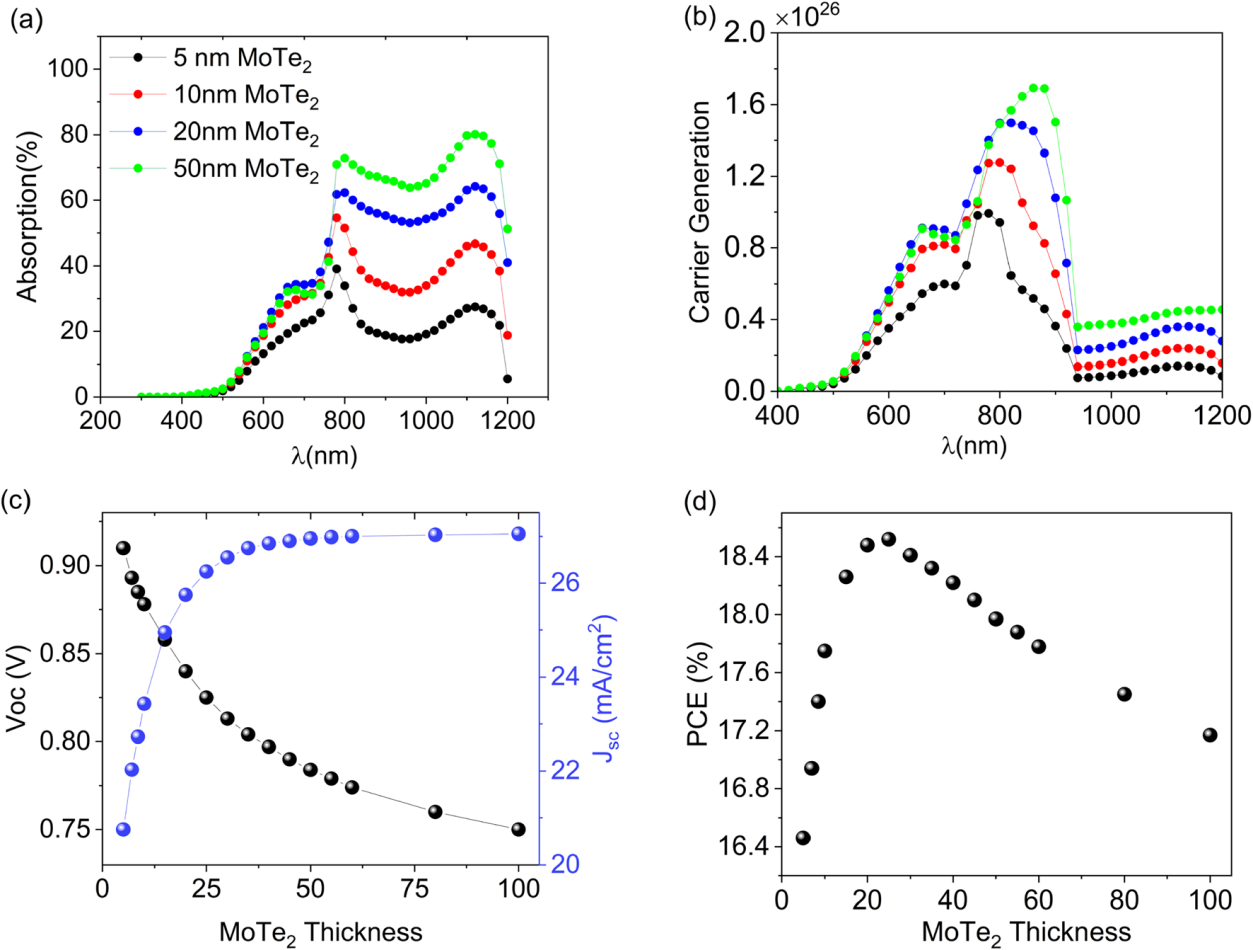
The multijunction PSC performance dependance on MoTe_2_ thickness. (**a**) The absorption spectra of the MoTe_2_ film with different thicknesses. (**b**) The carrier generation inside the different thicknesses of the MoTe_2_ layer. (**c**) The black and blue curves represent the dependence of V_OC_ and J_SC_ on MoTe_2_ thickness, respectively. (**d**) The dependance of PCE on MoTe_2_ thickness.

To achieve the multijunction PSC peak performance, an analysis of the cell performance dependence on the MoTe_2_ thickness has been carried out, while other input parameters in Table 1 are left unchanged. According to Fig. 3, the absorption, carrier generation, and photovoltaic parameters of the cell change as the MoTe_2_ thickness increase from 5 to 100 nm. Figure 3a exhibits the absorption spectra of four different thicknesses of the MoTe_2_ layer inside the multijunction PSC. As expected, the thicker the MoTe_2_ layer, the more light absorption in the MoTe_2_ layer. However, the light absorption rate becomes slower as the MoTe_2_ thickness increases, until it reaches saturation at a certain thickness. Even though too much light is absorbed by the MoTe_2_ at the longer wavelengths around 1100 nm, the carrier generation is poor at such wavelengths, as illustrate in Fig. 3b. This can be ascribed to resonant cavity effect and interference that play a role in absorption spectra, but do not exert any influence on carrier generation. As shown in Fig. 3c,d, photovoltaic parameters of the cell, PCE, J_sc_, V_oc_, and FF vary with the MoTe_2_ thickness. With increasing MoTe_2_ layer thickness, the J_sc_ gradually increases until it reaches a point of saturation. Conversely, the V_oc_ reduces as the MoTe_2_ thickness increases. The V_oc_ initially experiences a quick decrease and then the decrease rate becomes slower with the increase of MoTe_2_ thickness. The decreasing V_oc_ value can be assigned to an increase in charge carrier recombination in the thicker absorbing layer and to the increased series resistance^44^. When the absorbing layer thickness is smaller than the carrier diffusion length, the carrier recombination rate significantly diminishes, resulting in a sharp increase in V_oc_. On the other hand, after a distance as much as carrier diffusion length, a V_oc_ reduction occurs arising from the carrier recombination increase. Also, it is worth noting that the FF parameter has a negligible dependency on the MoTe_2_ thickness. Consequently, as indicated in the Fig. 3d, the PCE initially undergoes a relatively intense increase in the response to both V_oc_ and J_SC_ sharp changes in the thinner MoTe_2_ thicknesses and then reaches a maximum (~ 18.52%) at the MoTe_2_ thickness of 25 nm, and subsequently, it drops off as the J_sc_ increase is saturated.

**Figure 4:**
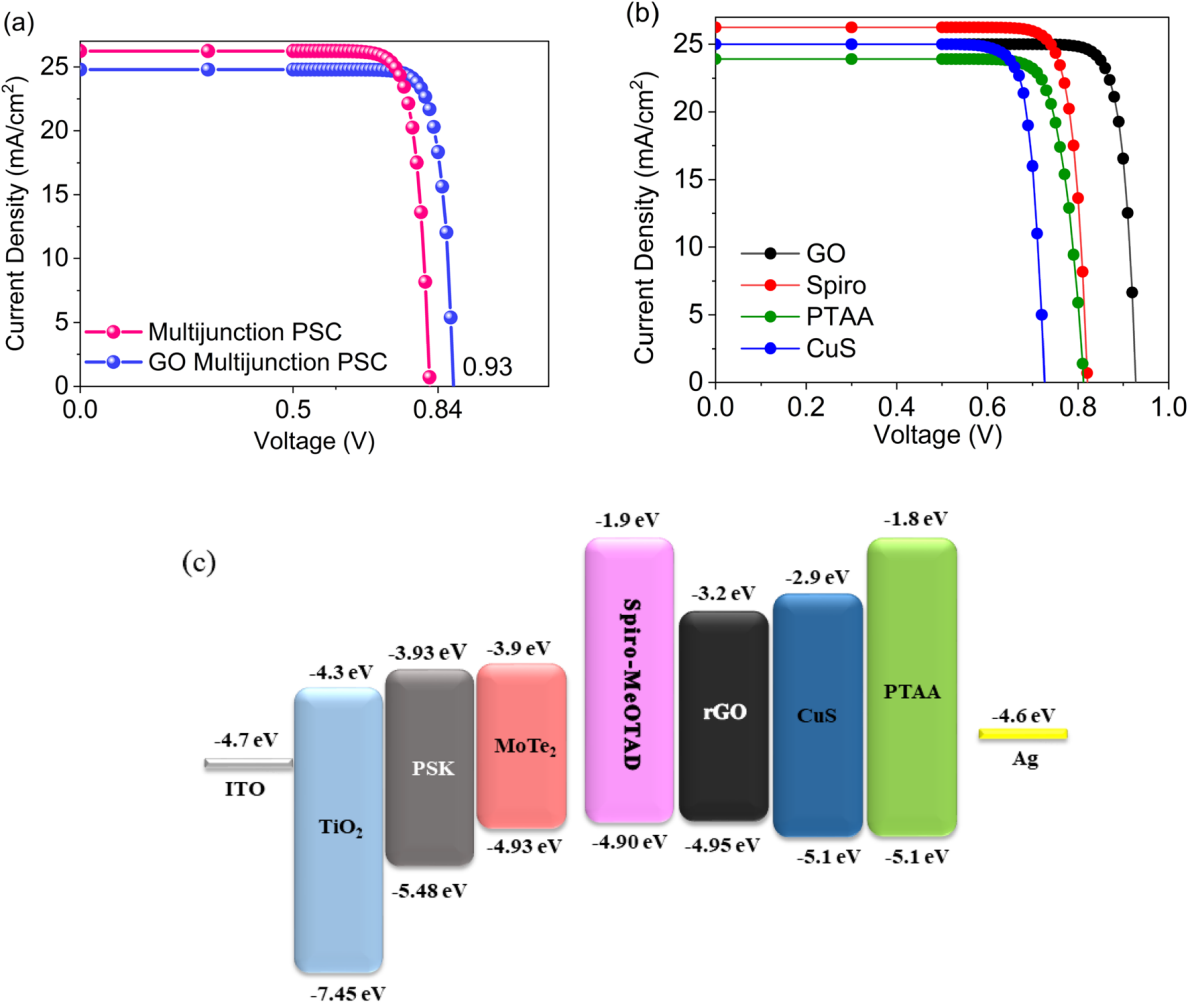
The multijunction PSC performance with and without rGO. (**a**) Current density–voltage (J–V) curve of the multijunction PSC performance with sprio and without rGO HTLs. (**b**) Current density–voltage (J–V) curve of the multijunction PSC with different HTL including Spiro, PTAA, rGO, and CuS. (**c**) Band alignment between the layers of multijunction PSC and the proposed HTLs.

In order to compensate the destructive effect of parallelly stacking low and high bandgap materials, we replace the spiro layer with a 60 nm rGO layer to improve the carrier transfer. Arguably, graphene oxide (GO) and rGO can provide multi-benefit to PSCs, namely, the improvement of stability, electrical, and thermal conductivity^45^. Hence, the materials were widely used for different functions in PSCs such as carrier transport layers, interlayers, and transparent conductive oxides. Here, the GO layer is selected to insert as a HTL due to its well-aligned bandstructure with the adjacent layers band edges. The electronic energy band parameters of rGO are obtained from the Ref^46^. As illustrated in the Fig. 4a, the utility of rGO as HTL notably improves both FF and Voc up to 0.89 and 0.928, respectively, in the comparison with the multijunction PSC without the rGO layer. Consequently, it yields a PCE as high as 20.32, around 1.77% larger than the multijunction PSC with spiro HTL. The significant improvement of photovoltaic performance in rGO-based multijunction PSC is devoted to more efficient charge transport and better energy band alignment, alongside a reduction in the increased series resistance owing to the expected charge recombination reduction at the interface.

Figure 4b compares the photovoltaic performance of multijunction PSC with different HTLs including Spiro, PTAA, rGO, and CuS materials. The input parameters of these materials are tabulated in Table 2. The rGO layer acts as HTL better than other materials due to its high hole mobility^47^, along with a nice band alignment with MoTe_2_. Conversely, CuS is not well energetically aligned with MoTe_2_, leading a V_OC_ reduction. The band diagram of multijunction PSC with different HTLs is shown in the Fig. 4c.

**Table 2:** The input simulation parameters.

Parameters	rGO	PTAA	CuS	Spiro
ε_r_	25	3	2.5	3
N_c_ (cm^−3^)	2.5 × 10^18^	2.5 × 10^18^	2.5 × 10^18^	2.5 × 10^18^
N_v_ (cm^−3^)	1.8 × 10^19^	1.8 × 10^19^	1.8 × 10^19^	1.8 × 10^19^
µ_n_/µ_p_ (cm^2^/VS)	372/372	5 × 10^–3^/5 × 10^–3^	1 × 10^–3^/1 × 10^–3^	10^–3^/10^–3^
χ (eV)	3.2	1.8	2.9	1.9
E_g_ (eV)	1.75	3.3	2.2	3.0
NA (cm^−3^)	5 × 10^19^	5 × 10^19^	5 × 10^19^	5 × 10^19^
ND (cm^−3^)	–	–	–	–
τ_n_/τ_p_ (ns)	3/3	0.48/0.48	5/5	7.1/7.1

## Conclusion

In summary, the significant successes achieved in multijunction (tandem) PSCs have intensified scientific efforts to address existing challenges and enhance their current performance. In this direction, we designed and proposed an n–i–p multijunction perovskite solar cell made of ITO/TiO_2_/CH_3_NH_3_PbI_3_/MoTe_2_/Spiro-OMeTAD/Ag layers, including two absorbers, the CH_3_NH_3_PbI_3_ and MoTe_2_, with cascaded bandgaps to absorb a wider solar spectrum. The MoTe_2_ layer with a bandgap around 1 eV enables us to harvest photons with energies smaller than the perovskite bandgap. The calculated results show an appreciable increase in the perovskite solar cell efficiency originating from the short circuit current, compared to the cell without MoTe_2_. Nevertheless, in a sharp contrast to the short circuit current, stacking the absorbers with different bandgaps has led to a fall in the open circuit voltage because of hole transport deterioration in the absorbing area. In order to alleviate the unavoidable issue, we inserted a graphene oxide layer with a thickness of 1.5 nm. Consequently, we observed that the open circuit voltage increases as much as 0.1 eV, leading to an efficiency improvement from 18.52% to 20.32%. Both MoTe_2_ and graphene oxide layers energy bandstructure are perfectly matched with their nearby layers band edges, allowing for achieving a high performance. It is also important to mention that the MoTe_2_ and graphene oxide layers chosen in this research have been experimentally utilized for various functions, such as stability improvement, transport layers, etc.

## Data Availability

The datasets used and/or analyzed during the current study are available from the corresponding author on reasonable request.
